# Clinical Characteristics of an Internet-Based Cohort of Patient-Reported Diagnosis of Granulomatosis With Polyangiitis and Microscopic Polyangiitis: Observational Study

**DOI:** 10.2196/17231

**Published:** 2020-07-20

**Authors:** Jason Michael Springer, Tanaz A Kermani, Antoine Sreih, Dianne G Shaw, Kalen Young, Cristina M Burroughs, Peter A Merkel

**Affiliations:** 1 University of Kansas Medical Center Kansas City, KS United States; 2 Division of Rheumatology Department of Medicine University of California Santa Monica, CA United States; 3 Division of Rheumatology Department of Medicine University of Pennsylvania Philadelphia, PA United States; 4 Vasculitis Foundation Kansas City, MO United States; 5 Health Informatics Institute University of South Florida Tampa, FL United States; 6 Division of Clinical Epidemiology Department of Biostatistics, Epidemiology, and Informatics University of Pennsylvania Philadelphia, PA United States

**Keywords:** granulomatosis with polyangiitis, microscopic polyangiitis, vasculitis, patient-reported outcomes, patient registry, electronic health records, questionnaire, online cohort

## Abstract

**Background:**

Utilizing the traditional centers of excellence approach to conduct clinical trials involving rare diseases remains challenging. Patient-based registries have been shown to be both feasible and valid in several other diseases.

**Objective:**

This report outlines the clinical characteristics of a large internet registry cohort of participants with a self-reported diagnosis of granulomatosis with polyangiitis or microscopic polyangiitis.

**Methods:**

Patients with a self-reported diagnosis of granulomatosis with polyangiitis or microscopic polyangiitis in an internet-based prospective longitudinal cohort (from the Vasculitis Patient-Powered Research Network) were included. Data on symptoms, diagnostic testing, and treatment were collected using standardized questionnaires.

**Results:**

The study compared patients with granulomatosis with polyangiitis (n=762) and patients with microscopic polyangiitis (n=164). Of the cohort, 97.7% (904/925) reported the diagnosis had been confirmed by a physician. Compared to microscopic polyangiitis, patients with granulomatosis with polyangiitis reported significantly more ear, nose, and throat manifestations (granulomatosis with polyangiitis: 641/723, 88.7%; microscopic polyangiitis: 89/164, 54.3%; *z*=10.42, *P*<.001), fevers (granulomatosis with polyangiitis: 325/588, 55.3%; microscopic polyangiitis: 64/139, 46.0%; *z*=1.96, *P*=.05), joint involvement (granulomatosis with polyangiitis: 549/688, 79.8%; microscopic polyangiitis: 106/154, 68.8%; *z*=2.96, *P*=.003), and pulmonary involvement (granulomatosis with polyangiitis: 523/734, 71.3%; microscopic polyangiitis: 90/154, 58.4%; *z*=3.13, *P*=.002). Compared to microscopic polyangiitis, patients with granulomatosis with polyangiitis reported significantly less renal involvement (granulomatosis with polyangiitis: 457/743, 61.5%; microscopic polyangiitis: 135/163, 82.8%; *z*=–5.18, *P*<.001) and renal transplantation (granulomatosis with polyangiitis: 10/721, 1.4%; microscopic polyangiitis: 7/164, 4.3%; *z*=–2.43, *P*=.02). Antineutrophil cytoplasmic antibody positivity was reported in 94.2% (652/692) of patients with granulomatosis with polyangiitis and 96.1% (147/153) of patients with microscopic polyangiitis. A biopsy showing vasculitis was reported in 77.0% (562/730) of patients with granulomatosis with polyangiitis and 81.9% (131/160) of patients with microscopic polyangiitis.

**Conclusions:**

In this large, internet-based cohort of patients with a self-reported diagnosis of granulomatosis with polyangiitis or microscopic polyangiitis, disease manifestations were consistent with expectations for each type of vasculitis. Given the rarity of these and other vasculitides, conducting some types of research through internet-based registries may provide an efficient alternative to inperson, center-of-excellence clinical trials.

## Introduction

Granulomatosis with polyangiitis and microscopic polyangiitis are forms of antineutrophil cytoplasmic antibody–associated vasculitis that primarily target small arteries. These are rare diseases, with annual prevalence of granulomatosis with polyangiitis estimated from 24 to 160 per 1,000,000 and annual prevalence of microscopic polyangiitis estimated from 39 to 94 per 1,000,000 [1]. There are several challenges in conducting clinical trials involving rare diseases, including the need to involve multiple centers, high costs, and other logistical challenges. Novel methods for obtaining both meaningful and reliable data are needed. Multiple studies [2-6] of other diseases have proven the validity of patient-reported diagnoses and outcomes. The aims of this study were to describe the self-reported clinical features of patients with granulomatosis with polyangiitis and microscopic polyangiitis who participated in the Vasculitis Patient-Powered Research Network and to establish to what extent this internet-based cohort is representative of the general population of patients with these forms of antineutrophil cytoplasmic antibody–associated vasculitis.

## Methods

Established in 2014, the Vasculitis Patient-Powered Research Network is an international, internet-based prospective longitudinal registry of patient- or caregiver-reported information. The Vasculitis Patient-Powered Research Network was established as a partnership between the Vasculitis Clinical Research Network (a vasculitis research network) and the Vasculitis Foundation (the largest patient advocacy group for vasculitis). The network represents a collaboration among a variety of vasculitis stakeholders including patients, patient advocacy organizations, academic clinical investigators, expert clinicians, biomedical informaticians, methodologists, and funding organizations. Patient-partners are an integral part of team and are involved in strategically planning, developing, reviewing, and approving research studies. Patient-partners receive training in patient participation in research.

The Vasculitis Patient-Powered Research Network is the largest patient-based registry for primary systemic vasculitis with over 3000 patients enrolled to date; patients (or caregivers) consent to participate in studies and provide self-reported information longitudinally using the internet-based platform [7]. For this study, advertisement for recruitment was done via social media, the Vasculitis Foundation website, and flyers at national and regional vasculitis conferences.

Only patients with a self-reported diagnosis of granulomatosis with polyangiitis and microscopic polyangiitis were included in this study. Patients provided consent online and were enrolled between November 2014 and May 2019. The data were obtained via convenience (or opportunity) sampling methods. Using the internet-based patient portal, participants filled out standardized questionnaires (open survey, Multimedia Appendix 1) in English which requested information such as demographics (age, gender, ethnicity), signs and symptoms of vasculitis at the time of diagnosis, results of diagnostic studies performed (ie, laboratory testing, biopsies, imaging, etc), prescribed treatments, and outcomes. For each item, respondents could select *yes*, *no*, or *I don’t know* and were able to review or change previously answered questions. Responses that were left blank or where the answer was not known were excluded from the analysis. Patient participation was encouraged by sending email reminders to participants; however, no incentives were offered for completion of the questionnaires. Multiple entries by the same individual were prevented through the use of password-protected user log-ins.

This study was approved by the institutional review board of the University of South Florida. To address data completeness and compliance, the Vasculitis Patient-Powered Research Network operates a comprehensive data compliance strategy using a variety of tools and approaches. The data compliance reports are regularly monitored by the network and data managers of the Vasculitis Patient-Powered Research Network to identify emerging trends. If a participant has not completed all forms, a series of automated email reminders to the participant are triggered. After an initial, generic reminder email message has been sent, a form-specific email reminder is sent. This message specifies which forms are incomplete and also describes the scientific significance and need for the information requested by each form.

Data were analyzed to compare clinical manifestations and diagnostic testing of patients with granulomatosis with polyangiitis to those of patients with microscopic polyangiitis. Two-tailed *z* scores were performed for comparisons of proportions. Two-tailed independent *t* tests were used for comparisons of means and medians. *P* values≤.05 were considered significant.

## Results

### Participant Characteristics

#### Granulomatosis With Polyangiitis

**Figure 1:**
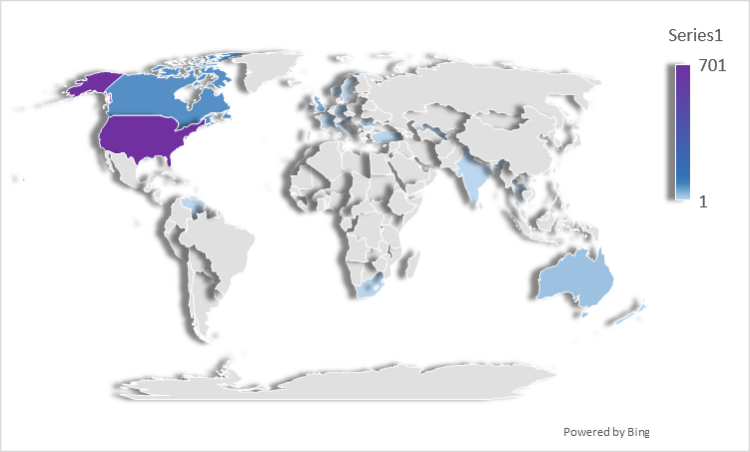
Heat maps showing geographic distribution of participants with granulomatosis with polyangiitis and microscopic polyangiitis internationally.

**Figure 2:**
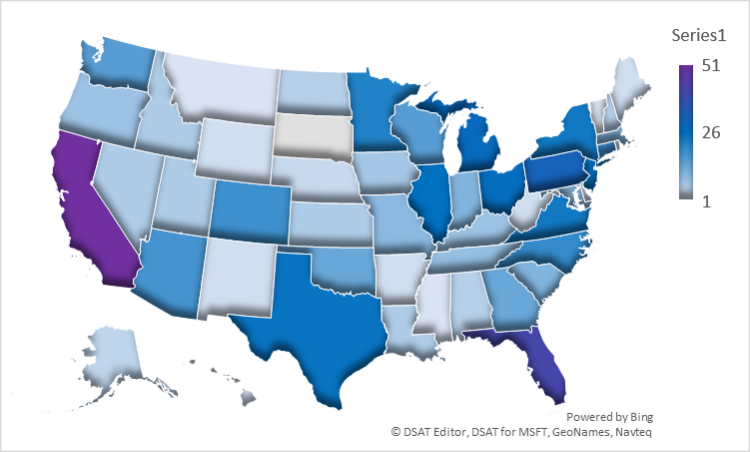
Heat maps showing geographic distribution of participants with granulomatosis with polyangiitis and microscopic polyangiitis within the United States of America.

A total of 762 participants reported a diagnosis of granulomatosis with polyangiitis; 518 (68.0%) were female and 244 (32.0%) were male. The median age of patients at the onset of symptoms was 45 (IQR 31-57) for 619 respondents, and the median age at diagnosis was 48 (IQR 35-57) for 683 respondents. Out of 761 respondents, 248 (32.6%) reported their disease as active, 465 (61.1%) reported their disease as being in remission, and 48 (6.3%) reported they were unsure. Respondents (648/727, 89.1%) reported seeing one or more of the following specialists: rheumatologist (528/727, 72.6%), nephrologist (202/727, 27.8%), pulmonologist (139/727, 19.1%), otolaryngologist (135/727, 18.6%), neurologist (29/727, 4.0%), immunologist (9/727, 1.2%), dermatologist (3/727, 0.4%). Respondents (720/762, 94.5%) reported their country of origin as the United States (572/720, 79.4%, of which state unreported: 72/572 and state reported: 500/572; northeastern states: 102/500, 20.4%; midwestern states: 132/500, 26.4%; southern states: 164/500, 32.8%; western states: 102/500, 20.4%), Canada (62/720, 8.6%), United Kingdom (36/720, 5.0%), Australia (18/720, 2.5%), or other (32/720, 4.4%) (Figure 1 and Figure 2).

#### Microscopic Polyangiitis

A total of 164 participants reported a diagnosis of microscopic polyangiitis; 133 (81.1%) were female and 31 (18.9%) were male. The median age of patients at the onset of symptoms was 52 (IQR 36-61) for 129 respondents, and the median age at diagnosis was 53 (IQR 41-62) for 158 respondents. Out of 160 respondents, 55 (34.4%) reported their disease as active, 96 (60.0%) reported their disease as being in remission, and 13 (8.1%) reported they were unsure. Respondents (141/160, 88.1%) reported seeing one or more of the following specialists: rheumatologist (95/160, 59.4%), nephrologist (85/160, 53.1%), pulmonologist (28/160, 17.5%), neurologist (6/160, 3.8%), immunologist (3/160, 1.9%), dermatologist (2/160, 1.2%) and otolaryngologist (1/160, 0.6%). Respondents (150/164, 91.5%) reported their country of origin as the United States (121/150, 80.6%, of which state unreported: 12/121 and state reported: 109/121; northeastern states: 15/109, 13.8%; midwestern states: 30/109, 27.6%; southern states: 31/109, 28.4%; western states: 33/109, 30.3%), Canada (10/150, 6.6%), United Kingdom (8/150, 5.3%), Australia (5/150, 3.3%), or other (6/150, 4.0%)

### Self-Reported Manifestations

In patients with granulomatosis with polyangiitis, the most common self-reported manifestations were nasal or sinus in 82.9% (600/723), joint pain in 79.8% (549/688), pulmonary in 71.3% (523/734), peripheral nerve in 62.8% (411/654), and renal in 61.5% (457/743) of respondents. Pulmonary–renal syndrome (diffuse alveolar hemorrhage in combination with renal disease) was reported by 26.9% (184/684) of participants. Venous thromboembolisms were reported by 13.7% (96/701) of respondents.

In patients with microscopic polyangiitis, the most common self-reported manifestations were renal in 82.8% (135/163), joint pain in 68.8% (106/154), peripheral nerve in 64.6% (95/147), nasal or sinus in 61.0% (89/146), rash in 59.6% (93/156), and pulmonary in 58% (90/154) of respondents. Pulmonary–renal syndrome was reported by 29% (47/161). Venous thromboembolism was reported in 13.6% (21/154) of respondents. Patients also reported isolated renal manifestations without other organ manifestations (14/164, 8.5%), although constitutional symptoms such as myalgia (1/164, 0.6%), fever (2/164, 1.2%), and weight loss (6/164, 3.7%) were reported.

### Diagnostic Testing

Confirmation of diagnosis by a physician was reported by 97.5% (742/761) of respondents with granulomatosis with polyangiitis. Patients with granulomatosis with polyangiitis underwent antineutrophil cytoplasmic antibody testing (652/692, 94.2%), laboratory testing (566/762, 74.3%), biopsy (475/762, 62.3%), and imaging (292/762, 38.3%) for diagnosis; symptom-based diagnosis was also reported (533/762, 69.9%). Out of 730 respondents, 562 (77.0%) reported having a biopsy showing vasculitis at some point, of which the biopsy sites included kidney (283/562, 50.4%), lung (192/562, 34.2%), nasal or sinus (146/562, 26.0%), skin (70/562, 12.5%), nerve (10/562, 1.8%), artery (7/562, 1.2%), and other sites (36/562, 6.4%).

Confirmation of diagnosis by a physician was reported by 98.8% (162/164) of respondents with microscopic polyangiitis. Patients with microscopic polyangiitis underwent antineutrophil cytoplasmic antibody testing (147/153, 96.1%), laboratory testing (129/164, 78.7%), biopsy (126/164, 76.8%), and imaging (69/164, 42.1%) for diagnosis; symptom-based diagnosis was also reported (110/164, 67.1%). Of 160 respondents, 131 (81.9%) reported having a biopsy showing vasculitis at some point, of which the biopsy sites included kidney (103/160, 78.6%), lung (20/160, 15.3%), skin (15/160, 11.5%), nerve (6/160, 4.6%), nasal or sinus (4/160, 3.1%), artery (3/160, 2.3%), and other sites (2/160, 1.5%). Additional diagnostic testing information can be found in Multimedia Appendix 2.

### Medications

For respondents with granulomatosis, the most commonly used medications included glucocorticoids (707/762, 92.8%), cyclophosphamide (total: 439/762, 57.6%; oral: 313/762, 41.1%; intravenous: 226/762, 29.7%), sulfamethoxazole and trimethoprim (418/762, 54.9%), rituximab (396/762, 52.0%), methotrexate (354/762, 46.5%), azathioprine (311/762, 40.8%), mycophenolate (140/762, 18.4%), and intravenous immunoglobulin (38/762, 5.0%); 8.4% (64/762) reported receiving plasma exchange at some point. For respondents with microscopic granulomatosis, the most commonly used medications included glucocorticoids (151/161, 93.8%), rituximab (89/161, 55.3%), cyclophosphamide (total: 79/161, 49.1%; oral: 44/161, 27.3%; intravenous: 49/161, 30.4%), azathioprine in (79/161, 49.1%), sulfamethoxazole and trimethoprim (53/161, 32.9%), mycophenolate (39/161, 24.2%), methotrexate (34/161, 21.1%), and intravenous immunoglobulin (9/161, 5.6%); 9.9% (16/161) reported receiving plasma exchange at some point. Additional data on the medications used by patients with granulomatosis with polyangiitis and by patients with microscopic polyangiitis to treat their vasculitis can be found in Multimedia Appendix 3.

### Granulomatosis With Polyangiitis Versus Microscopic Polyangiitis

Compared to those with a self-reported diagnosis of microscopic polyangiitis, those with a self-reported diagnosis of granulomatosis with polyangiitis were younger at both onset of symptoms (45 years of age versus 52 years of age, t_746_=–2.46, *P*=.01) and diagnosis (48 years of age versus 53 years of age, t_839_=–2.60, *P*=.01). Patients with granulomatosis with polyangiitis reported more sinonasal disease (83.0% versus 61.0%, *z*=5.99, *P*<.001), hearing loss (48.3% versus 15.3%, *z*=7.28, *P*<.001), tracheal involvement (30.0% versus 9.8%, *z*=4.80, *P*<.001), pulmonary involvement (71.3% versus 58.4%, *z*=3.13, *P*=.002), eye involvement (54.2% versus 33.8%, *z*=3.14, *P*=.002), joint involvement (79.8% versus 68.8%, *z*=2.96, *P*=.003) and fevers (55.3% versus 46.0%, *z*=1.96, *P*=.050) than patients with microscopic polyangiitis; whereas, those with microscopic polyangiitis reported more renal involvement (82.8% versus 61.5%, *z*=–5.18, *P*<.001) and were more likely to have undergone a renal transplant (4.3% versus 1.4%, *z*=–2.43, *P*=.02) than patients with granulomatosis with polyangiitis (Table 1). There was no difference in the proportion of venous thromboembolism (13.7% versus 13.6%, *z*=0.02, *P*=.99), no difference in the percentage of patients reporting a positive antineutrophil cytoplasmic antibody test (94.2% versus 96.1%, *z*=–0.92, *P*=.36), and no difference in the percentage of patients reporting a diagnosis based on biopsy (77.0% versus 81.9%, *z*=–1.35, *P*=.18). There were more diagnoses from lung (34.2% versus 15.3%, *z*=4.23, *P*<.001) and nasal or sinus biopsies (26.0% versus 3.1%, *z*=5.74, *P*<.001) in participants with granulomatosis with polyangiitis than those in participants with microscopic polyangiitis. Conversely, there were more diagnoses from kidney biopsy in participants with microscopic polyangiitis (78.6% versus 50.4%, *z*=–5.87, *P*<.001) than those in participants with granulomatosis with polyangiitis. A biopsy was more likely to have been performed in participants with microscopic polyangiitis than in those with granulomatosis with polyangiitis (76.8% versus 62.3%, *z*=–3.53, *P*<.001). Oral cyclophosphamide (*z*=3.25, *P*=.001), methotrexate (*z*=5.92, *P*<.001), and sulfamethoxazole and trimethoprim (*z*=5.06, *P*<.001) were more commonly used by patients with granulomatosis with polyangiitis than by those with microscopic polyangiitis (Multimedia Appendix 3).

**Table 1:** Self-reported clinical manifestations.

Clinical Manifestation		Granulomatosis with polyangiitis		Microscopic polyangiitis					
		N^a^	n (%)		N^a^	n (%)		*Z* score	*P* value
Rash		698	401 (57.4)		156	93 (59.6)		–0.50	.62
Weight loss		690	400 (58.0)		151	86 (57.0)		0.23	.82
Fever		588	325 (55.3)		139	64 (46.0)		1.96	.05
Joint		688	549 (79.8)		154	106 (68.8)		2.96	.003
**Ear, nose, and throat**									
	Any	723	641 (88.7)		164	89 (54.3)		10.42	<.001
	Nasal/sinus symptoms	723	600 (83.0)		146	89 (61.0)		5.99	<.001
	Hearing loss	666	322 (48.3)		144	22 (15.3)		7.28	<.001
	Tracheal	610	183 (30.0)		133	13 (9.8)		4.80	<.001
**Pulmonary**									
	Any	734	523 (71.3)		154	90 (58.4)		3.13	.002
	Alveolar hemorrhage	700	282 (40.3)		161	58 (36.0)		1.00	.32
**Renal**									
	Any	743	457 (61.5)		163	135 (82.8)		–5.18	<.001
	Dialysis	721	73 (10.1)		163	18 (11.0)		–0.35	.73
	Renal transplant	721	10 (1.4)		164	7 (4.3)		–2.43	.02
Pulmonary–renal		684	184 (26.9)		161	47 (29.2)		–0.59	.56
Peripheral nerve		654	411 (62.8)		147	95 (64.6)		–0.40	.69
Gastrointestinal		683	14 (2.0)		147	4 (2.7)		–0.51	.61
Venous thromboembolism		701	96 (13.7)		154	21 (13.6)		0.02	.99
Pericardial		609	49 (8.0)		132	6 (4.6)		1.39	.16
Eye		609	330 (54.2)		151	51 (33.8)		3.14	.002

^a^The number of patients who responded yes or no (unknown and blank responses were excluded).

## Discussion

### Principal Findings

This study was conducted using exclusively patient-reported information. It is important to understand how patient-derived data may differ from those obtained through traditional physician reports used in observational cohorts and clinical trials. This is the first large, prospective cohort of patients with a self-reported diagnosis of granulomatosis with polyangiitis or microscopic polyangiitis that included detailed information from standardized forms to evaluate clinical manifestations, the results of diagnostic testing, and types of treatment. The participants were from across the United States, Canada, and from multiple other countries. An analysis [8] of this cohort found that more than 90% of patients met the 1990 American College of Rheumatology classification criteria for granulomatosis with polyangiitis and the Chapel Hill Consensus Conference definition of microscopic polyangiitis.

The type and distribution of clinical manifestations among patients in this cohort were similar to those reported for observational cohorts and in clinical trials [9-11]. Ear, nose, and throat; pulmonary; and renal manifestations were common in granulomatosis with polyangiitis, as expected, while renal and lung involvement were common in microscopic polyangiitis. The proportions of positive antineutrophil cytoplasmic antibody tests (94.2% and 96.1%) were similar to what would be expected and to what has been in the literature [12]. It has been recognized that there is a high risk of venous thromboembolism in antineutrophil cytoplasmic antibody–associated vasculitis, especially during active disease. There was a 14% prevalence of venous thromboembolism in the combined cohort which was similar to that reported in the literature [13,14].

The most common means of diagnosis reported by participants (symptom-based, laboratory testing, biopsy, and imaging) closely reflected what was emphasized by the 1990 American College of Rheumatology classification criteria for granulomatosis with polyangiitis and the Chapel Hill Consensus Conference definition of microscopic polyangiitis. Only 1% of participants were not sure of the means of their diagnosis, reflecting good insight and recall of participants regarding the basis for their original diagnosis.

There were also important differences between this cohort and center-based cohorts. First, there was a higher than expected prevalence of skin (57.4% and 59.6%) and peripheral nerve involvement (62.8% and 64.6%). This could reflect the nonspecificity of the queries where patients were asked to attribute manifestations to vasculitis. Patients may have had difficulty adjudicating whether their symptoms were caused by vasculitis or as a result of complications of therapy.

Ear, nose, and throat manifestations are a hallmark manifestation for granulomatosis with polyangiitis distinguishing it from microscopic polyangiitis. In a study [15] comparing physician-reported clinical characteristics of granulomatosis with polyangiitis and microscopic polyangiitis in observational cohorts versus randomized controlled trials, 19% patients with microscopic polyangiitis had ear, nose, and throat manifestations compared to 76% patients with granulomatosis with polyangiitis. In this study cohort, there was a statistically higher (*z*=10.42, *P*<.001) prevalence of these manifestations in granulomatosis with polyangiitis as expected, but there was also a higher than expected prevalence in microscopic polyangiitis with nasal-sinus manifestations being reported in 54.3% of patients. This may represent the nonspecificity of queries which included asking patients to attribute manifestations to vasculitis. Alternatively, it may in part also reflect misclassification in the medical community in which patients who test positive for myeloperoxidase antineutrophil cytoplasmic antibodies are classified as having microscopic polyangiitis; and only patients who test positive for proteinase 3 antineutrophil cytoplasmic antibodies are classified as having granulomatosis with polyangiitis.

As expected, patients with microscopic polyangiitis were significantly more likely (*z*=–5.18, *P*<.001) to have had renal involvement than patients with granulomatosis with polyangiitis. In the microscopic polyangiitis group, 8.5% patients had isolated renal manifestations (renal-limited vasculitis). Overall, the proportion of renal involvement was similar to that reported in the literature [12]; however, in earlier reports, up to one-third of those with antineutrophil cytoplasmic antibody–associated vasculitis with renal involvement went on to develop end-stage renal disease [16] which was higher than what was found in this study (granulomatosis with polyangiitis: 73/457, 16.0%; microscopic polyangiitis: 18/135, 13.3%). This could be related to the improvement of renal outcomes in granulomatosis with polyangiitis and microscopic polyangiitis patients over time reflecting the changes in diagnosis and management [16,17]. This could also reflect the fact that there was a possible bias as a result of less morbidity in participants from the online portal. Furthermore, this study enrolled patients with a clinical diagnosis of granulomatosis with polyangiitis or microscopic polyangiitis; patients who had isolated renal manifestations and part of the spectrum of antineutrophil cytoplasmic antibody–associated vasculitis could have been underrepresented, especially if they had not been clinically diagnosed with granulomatosis with polyangiitis or microscopic polyangiitis by their physicians. Patients with renal-limited disease who have end-stage renal disease may also no longer be followed or monitored for vasculitis, and therefore, may not have enrolled in the study.

### Strengths and Limitations

This study had several strengths. First, the study cohort is large for such rare diseases, and is geographically diverse. The data elements of interest were selected and designed by highly experienced investigators in this field and collected using standardized forms. Patient input was obtained at each stage of the process, especially on the design of the user interface for the Vasculitis Patient-Powered Research Network website.

This study had several limitations. First, direct physician confirmation of the diagnosis was not obtained as part of this study; however, based on the patient information that was provided, more than 90% of patients with granulomatosis with polyangiitis and microscopic polyangiitis met either the 1990 American College of Rheumatology Classification Criteria for granulomatosis with polyangiitis or the Chapel Hill Consensus Conference definition for microscopic polyangiitis [8]. Almost all of the participants reported physician confirmation of the diagnosis. In addition, the manifestations described by patients were similar to those reported in the literature. Second, questions that were left blank or for which the participant answered, “I don’t know” were not included in the analysis. This may represent a response bias that is common among studies using self-reported data; however, for most questions this represented a small percentage of responses. Third, bias sampling may have led to an overrepresentation of certain subgroups. For instance, there were more women than men (2:1 for granulomatosis with polyangiitis and 4:3 for microscopic polyangiitis), despite the sex ratio in the general population being close to 1 for both diseases [1]. Internet-based participation in many surveys and studies is more common among women [18-20]. Age may also have been a factor in participation in this internet-based cohort. This appears to be less of an issue in this study which includes patients from across the age spectrum, including individuals older than 70 years. Fourth, the mode of survey response (internet) may have prevented some patients who lack access or higher education and since many of the participants were recruited through the Vasculitis Foundation, and it may also represent a bias toward participants who are savvier about their disease.

### Conclusions

Granulomatosis with polyangiitis and microscopic polyangiitis are both rare (also known as orphan diseases), which makes clinical research for these disorders difficult and requires the use of multiple centers which can be financially and logistically challenging. Patient registries offer an alternative to the centers of excellence approach to conducting clinical research. The data reported herein for an internet-based cohort demonstrated the feasibility of such registries across broad geographic regions and the high level of comparability between an internet-based and traditional academic center-based participant populations. These data provide investigators and patients with confidence that internet-based patient-reported data are reliable and can be used to conduct novel, cost-efficient medical research. Such internet-based registries offer an advantage in capturing participant data from those who would not otherwise be able to participate in studies.

## Appendix

Multimedia Appendix 1 Vasculitis Patient-Powered Research Network online questionnaire forms for granulomatosis with polyangiitis and microscopic polyangiitis.

Multimedia Appendix 2 Patient-reported testing methods to establish a diagnosis of granulomatosis with polyangiitis or microscopic polyangiitis.

Multimedia Appendix 3 Patient-reported medications used to treat granulomatosis with polyangiitis and microscopic polyangiitis.
