# Supplementary material for: Clinical Characteristics of an Internet-Based Cohort of Patient-Reported Diagnosis of Granulomatosis With Polyangiitis and Microscopic Polyangiitis: Observational Study
Source: J Med Internet Res. 2020 Jul 20;22(7):e17231. doi: 10.2196/17231 (PMC7428147; doi:10.2196/17231)
Supplement: Multimedia Appendix 1 [file jmir_v22i7e17231_app1.docx]

| VPPRN Basic Disease Related Information Form | | | |
| --- | --- | --- | --- |
| 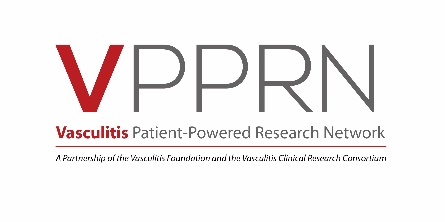 | ***Vasculitis Patient-Powered Research Network (VPPRN)*** | |  |
|  | | | |
| ***Tell us about your vasculitis*** | |  | |
| ***If you are a parent or caretaker, please complete these questions with the Patient’s information.*** | | | |
| ***Note****: all questions marked with * are required.* | | | |
|  | | | |
| ***Your Diagnosis*** | |  | |
| Approximately when did you have your first symptom of vasculitis?*  ⭘____ Month _____ Year (####) Month is optional; 4 digit Year is required  ⭘ Age at 1^st^ symptoms _________ years Numeric age is required  ⭘ I don’t know  Approximately when were you diagnosed with vasculitis? *  ⭘____ Month _____ Year(###) Month is optional; 4 digit Year is required  ⭘ Age at diagnosis _________ years Numeric age is required  ⭘ I don’t know  Did a doctor make the diagnosis of your vasculitis? *  ⭘ Yes  ⭘ No  ⭘ I don’t know  How was the diagnosis of vasculitis made in your case? **(check ALL that apply)** *  🞎 Based upon my symptoms  🞎 Laboratory testing (blood)  🞎 Radiology testing (X-rays, CT (or CAT) scans, MRIs)  🞎 Biopsy result  🞎 Angiogram (a test that uses special dye to take pictures of blood vessels)  🞎 DNA (gene) testing  🞎 I am not sure how my diagnosis of vasculitis was made  🞎 Other (please specify): ______________________________  What is the current state of your disease? **(please check only ONE)**  ⭘ Active disease  ⭘ Remission  ⭘ I don’t know   \| **VPPRN Disease-Specific Information Form for Patients with GPA or MPA** \| \| \| --- \| --- \| \| 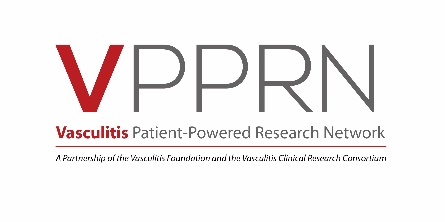 \| ***Vasculitis Patient-Powered Research Network (VPPRN)*** \| \|  \| \|   *Thank you for participating in the VPPRN. We would like to know more about your vasculitis. Answering the following questions will take less than 5 minutes but will help us understand better your condition and know how severe it is. (Please note not all patients experience every symptom described below. Remember to contact your treating physician if you have any new symptoms and for questions related to your individual treatment.)*  *Please note that the word* ***“related”*** *mentioned in the questions below means that you or your doctor believes is from active vasculitis or as a result of vasculitis.*  ***If you are a parent or caretaker, please complete these questions with the Patient’s information.***  1. Have you ever had problems with your nose **related** to your vasculitis (for example: nosebleeds, crusting, destruction of bone or cartilage within the nose) or problems with your sinuses **related** to your vasculitis (for example: sinusitis, congestion, pain, frequent infections)?*  ⭘ Yes  ⭘ No  ⭘ I don’t know  2. Have you ever had problems with your lungs **related** to your vasculitis (for example: coughing up blood, lung nodules/masses, abnormal chest X-ray or CT scan, need for lung biopsy)? *  ⭘ Yes  ⭘ No  ⭘ I don’t know  3. Have you ever been told there are problems with your kidneys **related** to your vasculitis (for example: blood in the urine, impaired kidney function/kidney damage, need for kidney biopsy)? *  ⭘ Yes  ⭘ No  ⭘ I don’t know  4. Have you ever had a positive blood test for antineutrophil cytoplasmic antibodies (ANCA)? *  ⭘ Yes  ⭘ No  ⭘ I don’t know  5. Have you ever had a biopsy (kidney, lung, nose, skin, etc…) that showed vasculitis? *  ⭘ Yes *[If yes selected, display question 5.1]*  ⭘ No  ⭘ I don’t know   - 1. Which organ was biopsied? ***(Please select ALL that apply)* ***   🞎 Skin  🞎 Lung  🞎 Kidney  🞎 Nerve  🞎 Nasal/Sinus  🞎 Artery or vein  🞎 Other:[Open text field]  🞎 I don’t know | | | |
| **VPPRN Baseline Medical History Form for Patients with GPA or MPA** | | | |
| 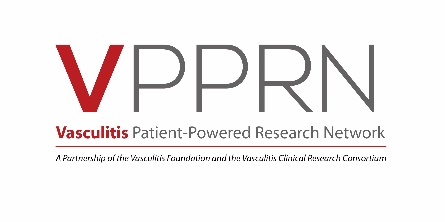 | ***Vasculitis Patient-Powered Research Network (VPPRN)*** | |  |
|  | | | |

*Thank you for participating in the VPPRN. We would like to know more about your vasculitis and the symptoms that you have experienced (at diagnosis* ***AND*** *over the course of your disease). Answering the following questions will take less than 10 minutes. Some of these questions may be similar to questions you have already answered.*

*It is very important that you complete this form. Your responses to these questions will help us better understand your condition and know how sever it is and will improve our greater understanding of the disease.*

*Please note the following when filling out this form:*

1. *Some of these questions may be similar to questions you have already answered.*
2. *Not all patients experience every symptom described below. Remember to contact your treating physician if you have any new symptoms and for questions related to your individual treatment.*
3. *The word* ***“related”*** *mentioned in the questions below means that you, or your doctor, believe is from active vasculitis or as a result of vasculitis.*

***If you are a parent or caretaker, please complete these questions with the Patient’s information.***

1. Have you ever lost weight (more than 10 lbs/4.5kg or more than 10% of your total weight) **related** to your vasculitis? *

⭘ Yes

⭘ No

⭘ I don’t know

1. Have you ever had a fever greater than 100.4 **°**F (38 **°** C) **related** to your vasculitis? *

⭘ Yes

⭘ No

⭘ I don’t know

1. Have you ever had severe joint pain or swelling **related** to your vasculitis? *

⭘ Yes

⭘ No

⭘ I don’t know

1. Have you ever had severe muscle pain **related** to your vasculitis? *

⭘ Yes

⭘ No

⭘ I don’t know

1. Have you ever had a rash (painful or painless red dots, ulcers, gangrene, mottled lacy pattern over the skin or portions of the extremities, nodules, or painful skin lesions) **related** to your vasculitis? *

⭘ Yes

⭘ No

⭘ I don’t know

1. Have you ever had oral ulcers or swelling in your gums **related** to your vasculitis? *

⭘ Yes

⭘ No

⭘ I don’t know

1. Have you ever had problems with your nose (nosebleeds, crusting, destruction of bone or cartilage within the nose) or problems with your sinuses (congestion, pain, frequent infections or sinusitis) **related** to your vasculitis? *

⭘ Yes

⭘ No

⭘ I don’t know

1. Have you ever lost hearing in one or both ears **related** to your vasculitis? *

⭘ Yes

⭘ No

⭘ I don’t know

1. Have you ever had swelling or narrowing (subglottic stenosis) in your windpipe (also known as trachea) **related** to your vasculitis? *

⭘ Yes

⭘ No

⭘ I don’t know

1. Have you ever had inflammation in one or both eyes that required treatment (such as conjunctivitis, uveitis, scleritis, retinal vasculitis) **related** to your vasculitis? *

⭘ Yes

⭘ No

⭘ I don’t know

1. Have you ever had problems with your lungs (lung nodules/masses, abnormal chest X-rays or CT scan, need for lung biopsy) **related** to your vasculitis? *

⭘ Yes

⭘ No

⭘ I don’t know

1. Have you ever coughed up blood or had bleeding in the lungs (also known as pulmonary hemorrhage or diffuse alveolar hemorrhage) **related** to your vasculitis? *

⭘ Yes

⭘ No

⭘ I don’t know

1. Have you ever been told there are problems with your kidneys (blood in the urine, impaired kidney function/kidney damage, need for kidney biopsy) **related** to your vasculitis? *

⭘ Yes

⭘ No

⭘ I don’t know

1. Have you ever needed dialysis because of kidney damage **related** to your vasculitis? *

⭘ Yes

⭘ No

⭘ I don’t know

1. Have you ever received a kidney transplant because of kidney damage **related** to your vasculitis? *

⭘ Yes

⭘ No

⭘ I don’t know

1. Have you had numbness, tingling, trouble moving your arms, hands, legs, or feet, or other forms of nerve damage **related** to your vasculitis?*

⭘ Yes

⭘ No

⭘ I don’t know

1. Have you ever had inflammation of the heart lining (also known as pericarditis) or inflammation of the heart muscle (also known as myocarditis) **related** to your vasculitis? *

⭘ Yes

⭘ No

⭘ I don’t know

1. Have you ever had loss of blood supply to your intestines or perforation (a hole or rupture of the wall) of your intestines **related** to your vasculitis? *

⭘ Yes

⭘ No

⭘ I don’t know

1. Have you ever had had a blood clot (also known as DVT or PE) in your arms, legs or lungs **related** to your vasculitis? *

⭘ Yes

⭘ No

⭘ I don’t know

1. Have you ever had a positive blood test for antineutrophil cytoplasmic antibodies (ANCA)?

⭘ Yes

⭘ No

⭘ I don’t know

1. Please list any other symptom that you believe is **related** to your vasculitis?
   a-…………..
   b-…………..
   c-…………...

| **VPPRN Baseline Medication Form** | | |
| --- | --- | --- |
| 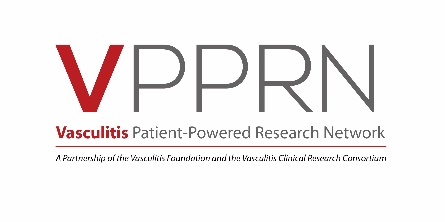 | ***Vasculitis Patient-Powered Research Network (VPPRN)*** |  |

| **Your Medications** |
| --- |
| Which medications have you **EVER** taken for the **treatment of your vasculitis (not for treatment of other side effects of vasculitis)**? ***Please check ALL that apply****  ❑ I have never taken any medications to treat my vasculitis  ❑ Aczone (Dapsone)  ❑ Arava (Leflunomide)  ❑ Aspirin  ❑ Asthma inhalers (for example, Albuterol, Pulmicort, Fluticasone inhaler)  ❑ Bactrim[Sulfamethoxazole/Trimethoprim]  ❑ Cellcept, Myfortic (Mycophenolate)  ❑ Colcrys (Colchicine)  ❑ Cytoxan (Cyclophosphamide)- **Intravenous (IV) Administration**  ❑ Cytoxan (Cyclophosphamide)-**Oral Administration (by mouth)**  ❑ Enbrel (Etanercept)  ❑ Humira (Adalimumab)  ❑ Hydrea (Hydroxyurea)  ❑ Imuran (Azathioprine)  ❑ Intravenous immunoglobulins (IVIg)  ❑ Intron-A or Roferon-A (Interferon alfa)  ❑ Methotrexate (Rheumatrex)  ❑ Orencia (Abatacept)  ❑ Plasmapheresis  ❑ Plavix (Clopidogrel)  ❑ Prednisone, prednisolone, or other glucocorticoid (“steroids”)  ❑ Remicade (Infliximab)  ❑ Respbid/Theo-24/Theolair (Theophylline)  ❑ Rituxan (Rituximab)  ❑ Sandimmune, Neoral (Cyclopsorine)  ❑ Singulair (Montelukast) or Accolate (Zafirlucast)  ❑ Xolair (Omalizumab)  ❑ Zocor (Simvastatin), Lipitor (atorvastatin), Pravachol (Pravastatin), Crestor (rosuvastatin) or any other “statin”  ❑ Experimental agent (please specify): ____________  ❑ Other ____________[add medication]  ❑ Other ____________[add medication]  ❑ Other ____________[add medication]  ❑ I don’t know  Which medications are you **CURRENTLY** taking for the **treatment of your vasculitis (not for treatment of other side effects of vasculitis)**? ***Please check ALL that apply****  ❑ I am not currently taking any medications to treat my vasculitis  ❑ Aczone (Dapsone)  ❑ Arava (Leflunomide)  ❑ Aspirin  ❑ Asthma inhalers (for example, Albuterol, Pulmicort, Fluticasone inhaler)  ❑ Bactrim[Sulfamethoxazole/Trimethoprim]  ❑ Cellcept, Myfortic (Mycophenolate)  ❑ Colcrys (Colchicine)  ❑ Cytoxan (Cyclophosphamide)- **Intravenous (IV) Administration**  ❑ Cytoxan (Cyclophosphamide)-**Oral Administration (by mouth)**  ❑ Enbrel (Etanercept)  ❑ Humira (Adalimumab)  ❑ Hydrea (Hydroxyurea)  ❑ Imuran (Azathioprine)  ❑ Intravenous immunoglobulins (IVIg)  ❑ Intron-A or Roferon-A (Interferon alfa)  ❑ Methotrexate (Rheumatrex)  ❑ Orencia (Abatacept)  ❑ Plasmapheresis  ❑ Plavix (Clopidogrel)  ❑ Prednisone, prednisolone, or other glucocorticoid (“steroids”)  **If user selects “** **Prednisone, prednisolone, or other glucocorticoid (“steroids”)”:**  Approximately what dose are you currently taking?  ___ ___. ___ mg  ⭘ Everyday ⭘ Every Other Day ⭘ Every Week ⭘ Every Other Week  ⭘ I don’t know  ❑ Remicade (Infliximab)  ❑ Respbid/Theo-24/Theolair (Theophylline)  ❑ Rituxan (Rituximab)  ❑ Sandimmune, Neoral (Cyclopsorine)  ❑ Singulair (Montelukast) or Accolate (Zafirlucast)  ❑ Xolair (Omalizumab)  ❑ Zocor (Simvastatin), Lipitor (atorvastatin), Pravachol (Pravastatin), Crestor (rosuvastatin) or any other “statin”  ❑ Experimental agent (please specify): ____________  ❑ Other ____________[add medication]  ❑ Other ____________[add medication]  ❑ Other ____________[add medication]  ❑ I don’t know  ***For each medication option selected under “current medications”, please display the following:***  Approximately when did you start taking this medication?*  _______ Month _________ Year  **(####) [Month is optional to provide; Year is required]**  ⭘ I don’t know   \| **VPPRN Follow-up Medication Form** \| \| \| \| --- \| --- \| --- \| \| 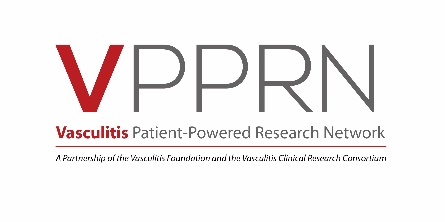 \| ***Vasculitis Patient-Powered Research Network (VPPRN)*** \|  \| **Your Medications** \| \| --- \| \| Which medications are you **CURRENTLY** taking for the **treatment of your vasculitis (not for treatment of other side effects of vasculitis)**? ***Please check ALL that apply****  ❑ I am not currently taking any medications to treat my vasculitis  ❑ Aczone (Dapsone)  ❑ Arava (Leflunomide)  ❑ Aspirin  ❑ Asthma inhalers (for example, Albuterol, Pulmicort, Fluticasone inhaler)  ❑ Bactrim[Sulfamethoxazole/Trimethoprim]  ❑ Cellcept, Myfortic (Mycophenolate)  ❑ Colcrys (Colchicine)  ❑ Cytoxan (Cyclophosphamide)- **Intravenous (IV) Administration**  ❑ Cytoxan (Cyclophosphamide)-**Oral Administration (by mouth)**  ❑ Enbrel (Etanercept)  ❑ Humira (Adalimumab)  ❑ Hydrea (Hydroxyurea)  ❑ Imuran (Azathioprine)  ❑ Intravenous immunoglobulins (IVIg)  ❑ Intron-A or Roferon-A (Interferon alfa)  ❑ Methotrexate (Rheumatrex)  ❑ Orencia (Abatacept)  ❑ Plasmapheresis  ❑ Plavix (Clopidogrel)  ❑ Prednisone, prednisolone, or other glucocorticoid (“steroids”)  **If user selects “** **Prednisone, prednisolone, or other glucocorticoid (“steroids”)”:**  Approximately what dose are you currently taking?  ___ ___. ___ mg  ⭘ Everyday ⭘ Every Other Day ⭘ Every Week ⭘ Every Other Week  ⭘ I don’t know  ❑ Remicade (Infliximab)  ❑ Respbid/Theo-24/Theolair (Theophylline)  ❑ Rituxan (Rituximab)  ❑ Sandimmune, Neoral (Cyclopsorine)  ❑ Singulair (Montelukast) or Accolate (Zafirlucast)  ❑ Xolair (Omalizumab)  ❑ Zocor (Simvastatin), Lipitor (atorvastatin), Pravachol (Pravastatin), Crestor (rosuvastatin) or any other “statin”  ❑ Experimental agent (please specify): ____________  ❑ Other ____________[add medication]  ❑ Other ____________[add medication]  ❑ Other ____________[add medication]  ❑ I don’t know  ***For each medication option, please display the following:***  Approximately when did you **start** taking this medication?*  _______ Month _________ Year  **(####) [Month is optional to provide; Year is required]**  ⭘ I don’t know  Have you **stopped** taking this medication?*  ⭘ Yes *[If user clicks on “Yes”, display Stop Date Question]*  ⭘ No  ⭘ I don’t know  Approximately when did you stop taking this medication?  **Stop Date:**  _______ Month _________ Year  **(####) [Month is optional to provide; Year is required]**  ⭘ I don’t know   \| **VPPRN Medical Team Information Form** \| \| \| \| --- \| --- \| --- \| \| 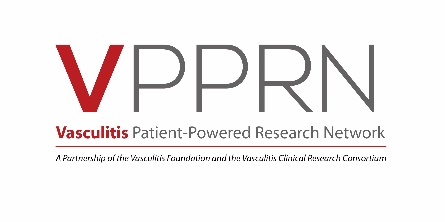 \| ***Vasculitis Patient-Powered Research Network (VPPRN)*** \| \| \| **Tell us about your healthcare team** \| \| --- \| \| *We would like to learn about the healthcare team that is responsible for your vasculitis care. Below you will be asked to provide information about your Physicians. Please provide information for the physicians that treat your vasculitis as well as your primary care physician (if different from the physician treating your vasculitis)*  ***If you are a parent or caretaker, please complete these questions with the patient’s information.*** \| \| ***Note****: all questions marked with * are required.*  **Veteran Status**  Have you ever received care, or are currently receiving care, at a United States Department of Veterans Affairs Medical facility?  ⭘ Yes  ⭘ No  ⭘ I don’t know  ⭘ I prefer not to respond  **Your Physician(s)**  What kind of doctor is responsible for your vasculitis care? *(****please select ALL that apply)*** *  ❑ I have never regularly seen a doctor for my vasculitis care  ❑ Primary care physician (family doctor, general practitioner, pediatrician)  ❑ Internist (Internal medicine doctor)  ❑ Rheumatologist  ❑ Nephrologist (kidney doctor)  ❑ Neurologist (nerve specialist)  ❑ Otolaryngologist (ear, nose, and throat doctor)  ❑ Pulmonologist (lung doctor, respirologist)  ❑ Dermatologist (skin specialist)  ❑ Other type of doctor:___[*open text*]_________  ❑ I don’t know  **Please tell us about your Physicians:[Collect Physician Contact Information for up to 6 Physicians]**  **Physician Information:**  Physician Name: _________________________________  Physician Type:  ⭘ Primary care physician (family doctor, general practitioner, pediatrician)  ⭘ Internist (Internal medicine doctor)  ⭘ Rheumatologist  ⭘ Nephrologist (kidney doctor)  ⭘ Neurologist (nerve specialist)  ⭘ Otolaryngologist (ear, nose, and throat doctor)  ⭘ Pulmonologist (lung doctor, respirologist)  ⭘ Dermatologist (skin specialist)  ⭘ Other type of doctor:___[*open text*]_________  ⭘ I don’t know  Practice/Clinic/Hospital Name (if known): __________________________________  Address line 1: __________________________________  Address line 2: __________________________________  Country: [Drop Down]  City: __________________________________  State: __________________________________  Zip or Postal code: __________________________________  Phone: __________________ Fax: _________________ E-mail: _____________________  How often do you see this doctor?  Once every _[numeric field]___ ⭘ Weeks ⭘ Months ⭘ Years \| \|  \| \| \| \| \| \| \| |
|  |
