# Supplementary material for: Clinical Characteristics of an Internet-Based Cohort of Patient-Reported Diagnosis of Granulomatosis With Polyangiitis and Microscopic Polyangiitis: Observational Study
Source: J Med Internet Res. 2020 Jul 20;22(7):e17231. doi: 10.2196/17231 (PMC7428147; doi:10.2196/17231)
Supplement: Multimedia Appendix 2 [file jmir_v22i7e17231_app2.docx]

| Diagnosis | GPA (%) ^a^ | MPA (%) ^a^ | P-value ^b^ |
| --- | --- | --- | --- |
| Physician-confirmed diagnosis | 742/761 (98%) | 162/164 (99%) | .32 |
| ***Means of diagnosis*** |  |  |  |
| Symptoms | 533/762 (70%) | 110/164 (67%) | .47 |
| Laboratory | 566/762 (74%) | 129/164 (79%) | .24 |
| Positive ANCA test | 652/692 (94%) | 147/153 (96%) | .36 |
| Radiology | 292/762 (38%) | 69/164 (42%) | .37 |
| Biopsy | 475/762 (62%) | 126/164 (77%) | < .001 |
| Biopsy showing vasculitis † | 562/730 (77%) | 131/160 (82%) | .18 |
| Skin † | 70/562 (12%) | 15/131 (11%) | .75 |
| Lung † | 192/562 (34%) | 20/131 (15%) | < .001 |
| Kidney † | 283/562 (50%) | 103/131 (79%) | < .001 |
| Nerve † | 10/562 (2%) | 6/131 (5%) | .06 |
| Nasal/sinus † | 146/562 (26%) | 4/131 (3%) | < .001 |
| Artery † | 7/562 (1%) | 3/131 (2%) | .37 |
| Other † | 36/562 (6%) ‡ | 2/131 (2%) | .03 |
| Angiogram | 15/762 (2%) | 7/164 (4%) | .08 |
| Not sure | 10/762 (1%) | 2/164 (1%) | .92 |
| Other | 28/762 (4%) | 4/164 (2%) | .43 |
|  |  |  |  |

GPA: granulomatosis with polyangiitis; MPA: microscopic polyangiitis

^a^ Denominator is the number of patients who responded Yes/No (response of “I don’t know” excluded).

^b^2-sided p-value for z-test. † Number of patients reporting biopsy. ‡ biopsies Include orbital/periorbital (10), salivary gland/mouth (7), subglottic/trachea (7), bone (3), ear (3), vagina/bladder (2), breast (1), lymph node (1), kidney mass (1), brain (1), muscle (1), pancreas (1), pituitary (1), liver (1), bowel (1). □ Biopsies includes muscle (2).
