# Supplementary material for: Clinical Characteristics of an Internet-Based Cohort of Patient-Reported Diagnosis of Granulomatosis With Polyangiitis and Microscopic Polyangiitis: Observational Study
Source: J Med Internet Res. 2020 Jul 20;22(7):e17231. doi: 10.2196/17231 (PMC7428147; doi:10.2196/17231)
Supplement: Multimedia Appendix 3 [file jmir_v22i7e17231_app3.docx]

| Medication | GPA (%) | MPA (%) | P-value |
| --- | --- | --- | --- |
| Glucocorticoids | 707/762 (93%) | 151/161 (94%) | .65 |
| Rituximab | 396/762 (52%) | 89/161 (55%) | .44 |
| Cyclophosphamide | 439/762 (58%) | 79/161 (49%) | .047 |
| Intravenous | 226/762 (30%) | 49/161 (30%) | .85 |
| Oral | 313/762 (41%) | 44/161 (27%) | .001 |
| Methotrexate | 354/762 (46%) | 34/161 (21%) | < .001 |
| Azathioprine | 311/762 (41%) | 79/161 (49%) | .054 |
| Mycophenolate | 140/762 (18%) | 39/161 (24%) | .09 |
| Leflunomide | 9/762 (1%) | 1/161 (0.6%) | .53 |
| Cyclosporine | 8/762 (1%) | 3/161 (2%) | .39 |
| Abatacept | 9/762 (1%) | 0/161 (0%) | .17 |
| Plasmapheresis | 64/762 (8%) | 16/161 (9%) | .53 |
| Intravenous Immunoglobulin | 38/762 (5%) | 9/161 (6%) | .75 |
| Sulfamethoxazole/Trimethoprim | 418/762 (55%) | 53/161 (33%) | < .001 |
| Dapsone | 30/762 (4%) | 8/161 (5%) | .55 |
|  |  |  |  |

GPA: granulomatosis with polyangiitis; MPA: microscopic polyangiitis
